# Polar Glycosylated and Lateral Non-Glycosylated Flagella from *Aeromonas* *hydrophila* Strain AH-1 (Serotype O11)

**DOI:** 10.3390/ijms161226097

**Published:** 2015-11-27

**Authors:** Kelly M. Fulton, Elena Mendoza-Barberá, Susan M. Twine, Juan M. Tomás, Susana Merino

**Affiliations:** 1 National Research Council, 100 Sussex Drive, Ottawa, ON K1A0R1, Canada; Kelly.Fulton@nrc-cnrc.gc.ca (K.M.F.); Susan.Twine@nrc-cnrc.gc.ca (S.M.T.); 2 Departamento de Microbiología, Facultad de Biología, Universidad de Barcelona, Diagonal 645, Barcelona 08071, Spain; elenademendoza@hotmail.com (E.M.-B.); smerino@ub.edu (S.M.)

**Keywords:** *O*-flagellin polar glycosylation, lateral flagellin non-glycosylated, adhesion, biofilm, immune stimulation

## Abstract

Polar and but not lateral flagellin proteins from *Aeromonas* *hydrophila* strain AH-1 (serotype O11) were found to be glycosylated. Top-down mass spectrometry studies of purified polar flagellins suggested the presence of a 403 Da glycan of mass. Bottom-up mass spectrometry studies showed the polar flagellin peptides to be modified with 403 Da glycans in *O*-linkage. The MS fragmentation pattern of this putative glycan was similar to that of pseudaminic acid derivative. Mutants lacking the biosynthesis of pseudaminic acid (*pseB* and *pseI* homologues) were unable to produce polar flagella but no changes were observed in lateral flagella by post-transcriptional regulation of the flagellin. Complementation was achieved by reintroduction of the wild-type *pseB* and *pseI*. We compared two pathogenic features (adhesion to eukaryotic cells and biofilm production) between the wild-type strain and two kinds of mutants: mutants lacking polar flagella glycosylation and lacking the O11-antigen lipopolysaccharide (LPS) but with unaltered polar flagella glycosylation. Results suggest that polar flagella glycosylation is extremely important for *A. hydrophila* AH-1 adhesion to Hep-2 cells and biofilm formation. In addition, we show the importance of the polar flagella glycosylation for immune stimulation of IL-8 production via toll-“like” receptor 5 (TLR5).

## 1. Introduction

Mesophilic *Aeromonas* spp. strains are important pathogens of humans and lower vertebrates, including amphibians, reptiles, and fish [1]. Infections produced by these strains in humans can be classified into two major groups: noninvasive disease such as gastroenteritis, and systemic illnesses [2]. Strains from *Aeromonas* *hydrophila*, *A. veronii* biovar *veronii*, or *sobria* are described as virulent for humans [3] and fish [4]; these strains are serologically related by their *O*-antigen lipopolysaccharide (LPS) (serotype O11). This has a known chemical structure containing *O*-polysaccharide chains of homogeneous chain length [5]. In addition, strains express a crystalline surface array protein with a molecular weight of *ca*. 52,000, which forms the S layer that lies peripheral to the cell wall [6]. The strains from this serotype are the most frequently isolated from septicemia caused by mesophilic *Aeromonas* spp. [2].

Flagella motility in *Aeromonas* represents an important advantage, allowing bacteria to move towards favorable conditions or avoid unfavorable environments, and it allows it to successfully compete with other microorganisms [7]. The mesophilic *Aeromonas* spp. constitutively express a single polar flagellum, and approximately 60% express numerous lateral flagella when grown in viscous environments or on surfaces [8,9]. Several studies have shown that both the polar and lateral flagella systems of the mesophilic *Aeromonas* spp. are involved in adherence to both biotic and abiotic surfaces, as well as in the biofilm formation [10].

One of the most common protein post-translational modifications is glycosylation, which for many years was thought to be a solely eukaryotic mechanism. More recently, many different protein glycosylation systems have been largely identified in all forms of life including prokaryotes. Carbohydrates are covalently attached to serine or threonine residues (*O-*glycosylation), or to asparagine residues (*N*-glycosylation). A recent review gives an overview of *O-*glycosylation in bacterial systems [11]. The available bacterial genomic information and bioinformatic analysis, together with functional analysis, has allowed the identification of many genes that participate in flagellin glycosylation, and also the definition of glycosylation pathways. These studies showed the large diversity in each bacterial species in the number of *O-*glycosylation genes involved and their location [12,13,14,15]. However, the diversity of structure and composition of glycans which modify flagellins from Gram-negative bacteria is restricted to certain species, or strains, as described in a recent review [16].

In the current study we show that polar but not lateral flagellins of *A. hydrophila* strain AH-1 from serotype O11 [16] are modified at multiple sites with putative glycans, which we propose to be pseudaminic acid-like moieties. We also show the requirement of glycosylation for polar flagella production. The *O*-antigen LPS or the flagella have been described as important molecules for bacterial adherence or biofilm formation in *Aeromonas* [17]. We recently characterized the O11 antigen LPS from the same strain *A. hydrophila* AH-1, and obtained mutants lacking this LPS structure [18]. This allowed us to evaluate the importance of both flagellin and LPS in adhesion to Hep-2 cells and biofilm formation. In addition, the importance of polar flagella glycosylation for immune stimulation of IL-8 production via toll-“like” receptor 5 (TLR5) was also evaluated.

## 2. Results

**Figure 1:**
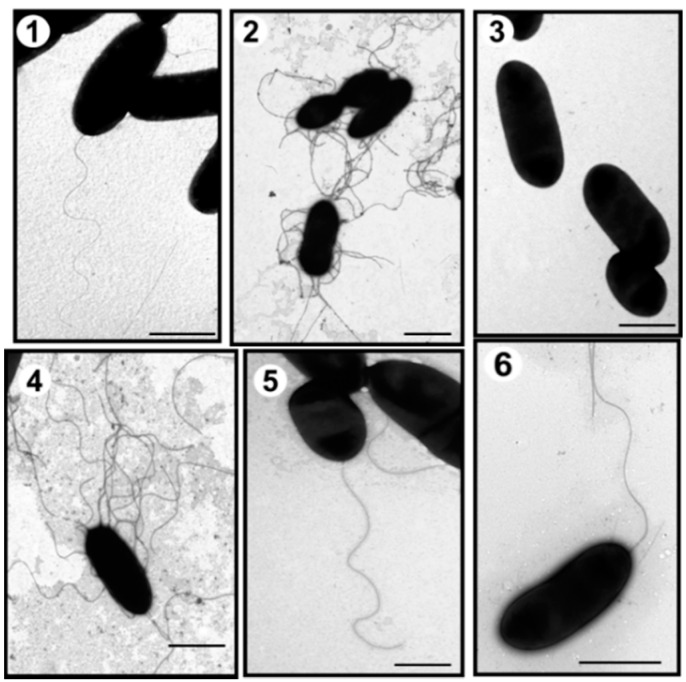
Transmission electron microscopy of *A. hydrophila* strains. AH-1 wild type grown in liquid media (TSB) (**1**) or in solid media (TSA) (**2**); AH-1Δ*pseI* mutant grown in TSB (**3**) or in TSA (**4**); AH-1Δ*pseI* mutant + pBAD-*pseI* grown in TSB (**5**), and AH-1Δ*rmlB* mutant grown in TSB (**6**). Bar = 1 μm.

The *A. hydrohila* AH-1 strain belongs to serotype O11 and is able to produce an S-layer [19]. This strain is motile in liquid medium (swimming) through expression of a polar flagellum and exhibits swarming behaviors in semisolid media through expression of lateral flagella (Figure 1).

DNA probes from polar flagella region 2 of *A. hydrophila* AH-3 [20] allowed identification of a clone from a cosmid genomic library of *A. hydrophila* AH-1. This clone allowed the DNA sequence of complete region 2 [21] of AH-1 to be obtained. This DNA sequence also allowed the isolation of a non-polar flagellated mutant AH-1ΔFlaB-J; this mutant was unable to swim, but able to swarm, suggesting expression of lateral flagella only. Subsequently, this mutant was used to isolate lateral flagella on this strain. DNA probes from the lateral flagella cluster of *A. hydrophila* AH-3 [22] allowed identification of a clone from the same cosmid genomic library of *A. hydrophila* AH-1 with the partial lateral flagella cluster of this strain. The partial DNA sequence of the AH-1 lateral flagella cluster allowed us to identify two lateral flagellins (LafA1 and A2) in this strain. This compares with a single lateral flagellin observed in strain AH-3 [22].

### 2.1. Mass Spectrometry Analyses of Wild-Type Lateral and Polar Flagellins

**Figure 2:**
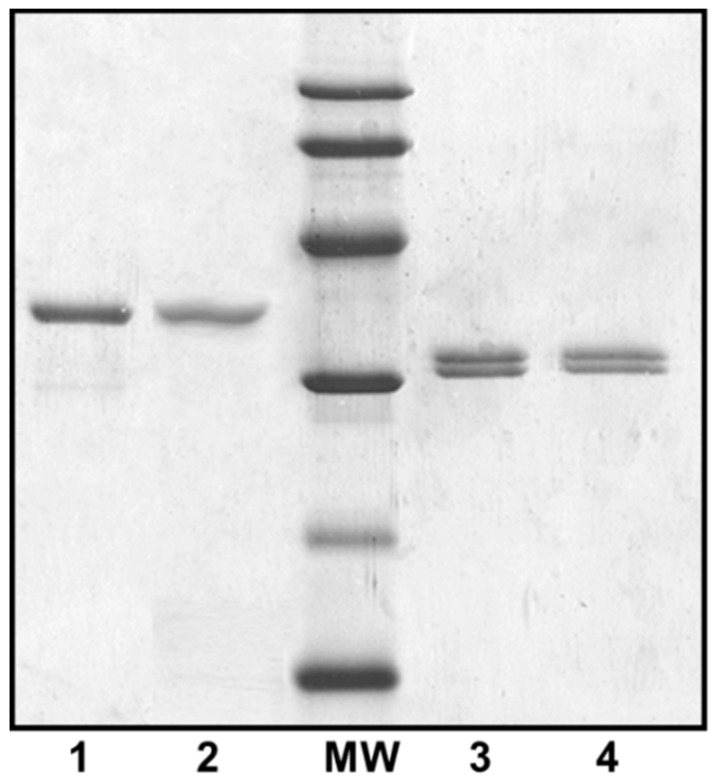
Purified polar and lateral flagellins from several *A. hydrophila* strains. 1, *A. hydrophila* AH-1 (wild-type) polar flagellin; 2, *A. hydrophila* AH-1∆*rmlB* mutant (no *O*-antigen LPS produced) polar flagellin; MW = molecular weight standard (14, 20, 30, 45, 60, and 94 kDa); 3, *A. hydrophila* AH-1Δ*flaB*-*J* (unable to produce polar flagellum) lateral flagellin; and 4, *A. hydrophila* AH-1∆*rmlB* mutant lateral flagellin.

Both polar and lateral flagellins were purified (Figure 2) and their intact mass profiles analyzed using LC–MS.

**Figure 3:**
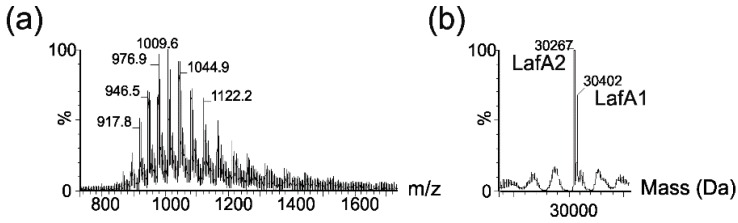
Electrospray mass spectrometry of intact lateral flagellin proteins from *Aeromonas hydrophila* AH-1. (**a**) Electrospray mass spectrum of intact lateral flagellin proteins showing a complex envelope of multiply charged protein ions; (**b**) The reconstructed molecular mass profile of the lateral flagellin resolves two masses at 30,267 and 30,402 Da, closely corresponding to the predicted mass of lateral flagellin proteins LafA1 and Laf A2.

The MS spectrum of a purified lateral flagellin showed a multiply charged ion envelope, as shown in Figure 3a, typical of protein. The spectrum was deconvoluted (Figure 3b) and showed two protein masses of 30,402 and 30,267 Da.

These masses corresponded almost exactly to the predicted masses of the lateral flagellin proteins, LafA1 (30,401 Da) and LafA2 (30,297 Da). In addition, nLC-MS/MS analyses of tryptic digests of polar flagellins showed 42% and 45% sequence coverage for LafA1 and LafA2, respectively. Manual inspection of the MS/MS data showed no evidence of glycan-related oxonium ions in peptide spectra. Taken together, these data strongly suggested that the flagellins were not post-translationally modified with carbohydrates, as had been reported with lateral flagellins of *A. hydrophila* AH-3 [23].

**Figure 4:**
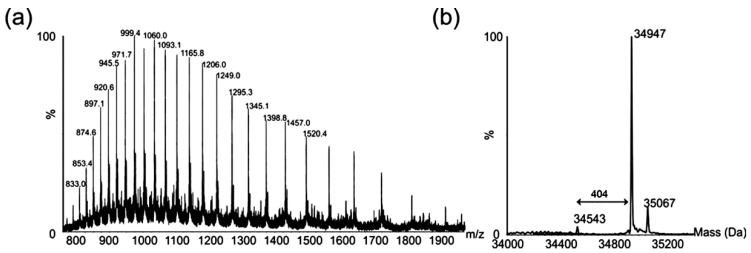
Electrospray mass spectrometry of intact polar flagellin proteins from *Aeromonas hydrophila* AH-1. (**a**) Electrospray mass spectrum of intact polar flagellin proteins showing a complex envelope of multiply charged protein ions; (**b**) The reconstructed molecular mass profile of these polar flagellins resolves three masses at 34,543, 34,947, and 35,067 Da.

LC-MS analysis of purified polar flagellins produced a characteristic multiply charged protein ion envelope (Figure 4a). When deconvoluted, a major mass of 34,947 Da was observed (Figure 4b).

The masses of the polar flagellin proteins predicted from the translated gene sequences are 31,413 and 31,361 Da, and neither mass was observed in the deconvoluted mass spectrum. The observed difference between the observed masses, highlighted in Figure 4b, suggests a mass difference between flagellins of 404 Da.

**Figure 5:**
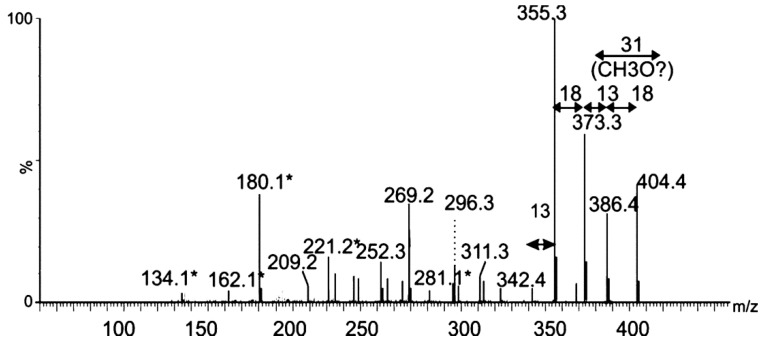
Mass spectrometry analysis of putative glycan oxonium ion. Tandem mass spectrum of singly charged ion at *m*/*z* 404 following feCID of intact polar flagellin protein showed consecutive losses of water and methyl groups from the parent ion. Subsequent losses from this ion gave rise to a fragmentation pattern similar to that observed with nonulosonic acid sugars. Daughter ions marked with an asterisk denote those fragment ions found in MS/MS spectra of pseudaminic acid. Figure legend: = 376 Da sugar; * = fragment ions common to pseudaminic acid.

To further investigate the nature of the protein modification, a tandem MS experiment was carried out on one multiply charged protein ion (*m*/*z* = 999.4). The resulting MS/MS spectrum was dominated by an intense ion at *m*/*z* = 404.4. An MS3 experiment on the ion observed at *m*/*z* = 404.4 produced an MS3 which showed consecutive losses of water and a methyl group from the parent ion (Figure 5).

The MS/MS spectrum did not contain any recognizable peptide-related ions, and the fragmentation pattern strongly suggested that this moiety was a glycan. Of note, many sugar fragment ions, observed at *m*/*z* *=* 134.1, 162.1, 180.13, 221.19, 281.1 (denoted with an asterisk in Figure 5), were also observed in the MS/MS spectrum of the flagellin-modifying sugar Pse5Ac7Ac9Ac, found in *A. caviae* [24]. Other fragment ions, observed at *m*/*z* = 342.4, 355.4 and 373.2, were a single *m*/*z* unit different from fragment ions observed in the MS/MS spectrum of Pse5Ac7Ac9Ac (Figure 4). From this data, the top-ranked plausible elemental formula was C_19_H_32_O_9_. These data, combined with the accurate mass analyses, suggest the base sugar is a pseudaminic acid-like sugar, with putative additions of two methyl groups and two molecules of water, and an unknown mass of 25 Da. Detailed structural analyses using NMR will be required to confirm this suggestion and the complete structure of this putative sugar moiety.

### 2.2. Tandem Mass Spectrometry Analyses of Proteolytic Digests of Polar Flagellin Proteins

**Figure 6:**
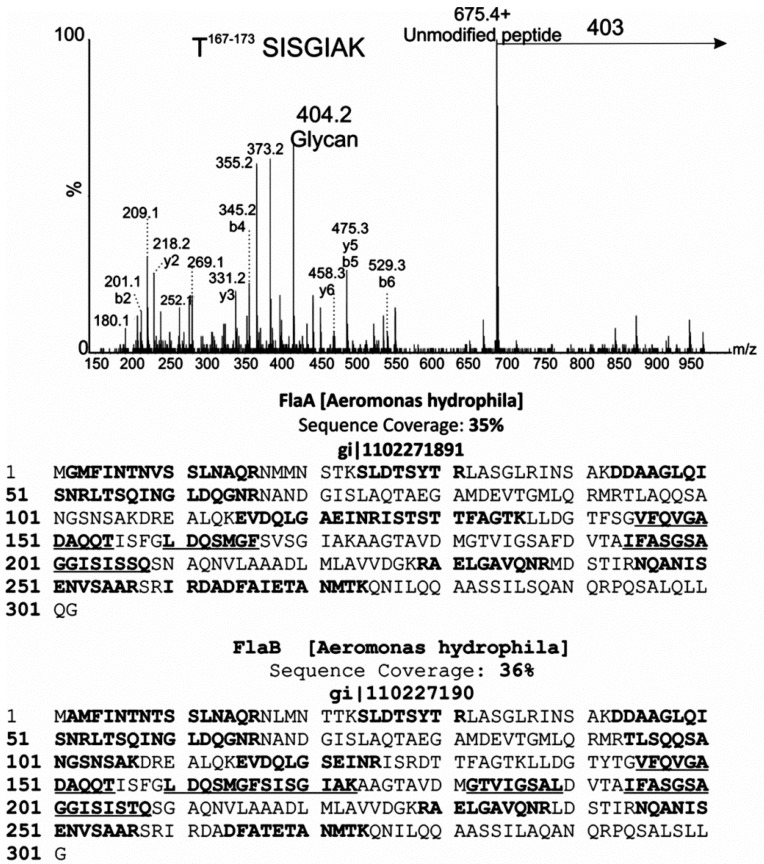
MS/MS analysis of glycopeptides from polar flagellar protein of *Aeromonas hydrophila* AH-1. nLC-MS/MS spectrum of the doubly protonated glycopeptide ion at *m*/*z* = 539.5. The MS/MS spectrum was dominated by an ion at *m*/*z* = 675.4, corresponding to the unmodified peptide ^167^SISGIAK^173^. A neutral loss of 403 Da was observed from the glycopeptide precursor, and a corresponding glycan oxonium ion observed at *m*/*z* = 404. Peptide type y and b ions are indicated, and were weak in comparison to glycan-related fragment ions, which are indicated by an asterisk. Peptide sequence coverage of lateral flagellins FlaA and B is shown below. In each case, bold indicates an identified non-modified peptide. Bold and underline indicates peptides that were *de novo* sequenced and modified with 403 Da glycan moiety.

nLC-MS/MS analyses of tryptic digests of polar flagellin preparation showed peptide sequences corresponding to FlaA and FlaB proteins giving 35% and 36% sequence coverage, respectively (Figure 6).

*De novo* sequencing of tryptic peptides showed many MS/MS spectra of high *m/z* peptide precursor ions, with very weak peptide type y or b fragment ions. Inspection of the protein sequence showed that the central variable region of the flagellin sequence was lacking tryptic cleavage sites, resulting in tryptic peptides likely too large to be readily sequenced by nLC-MS/MS. To overcome this challenge, the flagellin protein was treated with proteinase K for 15 min–16 h and the resulting peptides analyzed by nLC-MS/MS. From these data, a total of five non-overlapping glycopeptides were *de novo* sequenced, as indicated in Figure 6. All of the glycopeptides were observed to be modified with one or more glycan moieties of 403 Da, with the spectra dominated by an intense glycan oxonium ion at *m*/*z* = 404. This can be seen in Figure 6, which shows the MS/MS spectrum of the polar flagellin peptide ^167^SISGIAK^173^. Glycan fragment ions were observed in the low *m*/*z* region of the glycopeptide spectrum, indicated with an asterisk in Figure 6. In addition to the glycan oxonium ion at *m/z* *=* 404, glycan-related fragment ions were identical to those ions observed in the top-down fragmentation of the putative glycan. In all but one case, the glycopeptides corresponded to identical regions of FlaA and FlaB (except SISGIAK). The absence of asparagine residues on all sequenced glycopeptides suggests that the glycan is attached via *O*-linkage to serine or threonine residues.

### 2.3. Putative Pseudaminic Acid Biosynthetic Mutants

Due to the suggestion of a putative pseudaminic acid-like glycosylation of AH-1 polar flagella, genes in the biosynthetic pathway of this sugar were mutated. Using oligonucleotides 5′-TCCAGAAGGTTATCGCACT-3′ and 5′-GATGCTGGGAGCTATTACG-3′ with genomic DNA from strain AH-1, an internal DNA fragment (500 bp) corresponding to a pseB homologue was amplified [25]. Genome walking allowed the completion of the DNA sequence of pseB-C homologues in strain AH-1. The same approach using oligonucleotides 5′-CCTATACCGCTGACACC AT-3′ and 5′-TCACCACTTTTTCCTGACC-3′ was used to amplify an internal DNA fragment (679 bp) of pseI homologue [25], and completely sequence gene pseG-I homologues in this strain. Subsequently, in frame mutants AH-1∆pseB and AH-1∆pseI were constructed. Using TEM, these mutants were shown to be unable to produce polar flagellum but lateral flagella was unaffected under induced conditions by TEM (Figure 1 data shown for AH-1∆pseI). The introduction of the Aeromonas wild-type corresponding genes recovered the production of polar flagella in the mutants (data not shown).

### 2.4. Adhesion to HEp-2 Cells and Biofilm Formation

The role of polar flagella glycosylation, lateral flagella, and O11-antigen LPS in the adherence to eukaryotic cells was investigated. The adhesion of several mutants to cultured monolayers of HEp-2 cells was observed. The AH-1Δ*rmlB* mutant lacking O11-antigen LPS [18], but with expression of either polar or lateral flagella under induced conditions by TEM (Figure 1), showed no changes in flagellin molecular weight (Figure 2). Bacterial motility, when compared to wild type, was also unaffected, as was bacterial adherence to HEp-2 cells (Table 1).

By contract, mutants ∆*pseB* or *I* that lacked expression of flagella (polar and lateral for their grown in broth) showed a minimal adhesion values with a 86% reduction compared with the wild type. The same mutants expressed lateral flagella when grown in solid media, and showed an increase in their rate of adherence to Hep-2 cells (Table 1). The AH-1∆*rmlB* mutant lacking the O11-antigen LPS showed comparatively less reduction in adhesion (29%) compared with the reduced adhesion observed in the flagella mutants. In all the cases, complementation of mutants with the wild-type gene/s showed recovery of the wild-type adhesion values (Table 1). The results obtained in previous adhesion studies prompted us to study the biofilm production from the wild type and different mutant strains in a microtiter assay (Table 2).

**Table 1:** Adhesion of different *A. hydrophila* AH-1 serotype O11 strains to HEp-2 cells as described in Experimental Section. Values presented are mean ± SD from three independent experiments carried out in duplicate or triplicate (*n* = 6 or *n* = 9).

Strain and Main Characteristics	Mean No. of Bacteria	% Reduction
	HEp-2 Cell ± SD	in Adhesion ^a^
AH-1; wild-type serotype O11	21.4 ± 3.6	–
AH-1Δ*pseB* (flagella^−^) (O11^+^; flagella polar^−^/lateral^−^, grown in TSB)	2.7 ± 0.9	87 *
AH-1∆*pseB* (flagella^−^) (O11^+^; flagella polar^−^/lateral^+^, grown in TSA)	7.5 ± 1.8	65 *
AH-1∆*pseI* (flagella^−^) (O11^+^; flagella polar^−^/lateral^−^, grown in TSB)	2.9 ± 0.5	86 *
flagella polar^−^/lateral^−^, grown in TSA	8.1 ± 1.1	63 *
AH-1∆*pseB* + pBAD-*pseB* (flagella^−^) (O11^+^; flagella polar^+^/lateral^−^, grown in TSB)	19.8 ± 2.4	<8
flagella polar^+^/lateral^−^, grown in TSB	20.7 ± 2.0	<8
AH-1∆*rmlB* (O11^−^ mutant) (O11^−^; flagella^+^)	15.4 ± 2.6	29 *
AH-1∆*rmlB* + pBAD-*rmlB* (O11^+^; flagella^+^)	20.4 ± 3.2	<8

^a^ The level of adhesion of strain AH-1 was used as 100% value. Student’s *t-*test, *p* = 0.001 for adhesion values; * Values statistically different from the wild-type strain AH-1 level of adhesion. No significate differences were observed between the AH-1 level of adhesion when grown in liquid medium (polar flagella^+^, lateral flagella^−^) or solid medium (polar flagella^+^, lateral flagella^+^); −, lack of the corresponding structure; +, presence of the corresponding structure.

The wild-type strain grown on liquid media (no lateral flagella produced) showed biofilm formation ability with an average OD_570_ value of 1.43. Mutants lacking flagella grown in liquid or solid media (+ or − lateral flagella) are unable to form biofilms, with values <0.1, out of the range of detection for the assay (Table 2). The AH-1Δ*rmlB* mutant lacking the O11-antigen LPS showed an approximately 50% reduction in the ability to produce biofilm (0.78 average value *versus* 1.43 for wild type). In all cases, biofilm formations of the mutants were fully rescued by the introduction of the wild-type genes (Table 2). Mutants’ strains with only the plasmid vector alone show no differences in both studies.

**Table 2:** Biofilm values of several *A. hydrophila* AH-1 serotype O11 strains using the method of Pratt and Kolter as indicated in the Experimental Section. Values presented are mean ± SD from three independent experiments carried out in triplicate (*n* = 9).

Strain and Characteristics	Value (OD_570_)
AH-1, wild-type serotype O11	1.43 ± 0.15
AH-1∆pseB (flagella^−^) (O11^+^; flagella polar^−^/lateral^−^, grown in TSB)	<0.1
AH-1∆*pseB* (flagella^−^) (O11^+^; flagella polar^−^/lateral^+^, grown in TSA)	<0.1
AH-1Δ*pseI* (flagella^−^) (O11^+^; flagella polar^−^/lateral^−^, grown in TSB)	<0.1
AH-1∆*pseI* (flagella^−^) (O11^+^; flagella polar^−^/lateral^+^, grown in TSA)	<0.1
AH-1∆*pseB* + pBAD-*pseB* (flagella^−^) (O11^+^; flagella polar^+^/lateral^−^, grown in TSB)	1.37 ± 0.11
AH-1∆*pseI* + pBAD-*pseI* (flagella^−^) (O11^+^; flagella polar^+^/lateral^−^, grown in TSB)	1.39 ± 0.18
AH-1∆*rmlB* (O11^−^; flagella^+^)	0.78 ± 0.13
AH-1∆*rmlB* + pBAD-*rmlB*	1.41 ± 0.17

### 2.5. IL-8 Immune Stimulation

**Figure 7:**
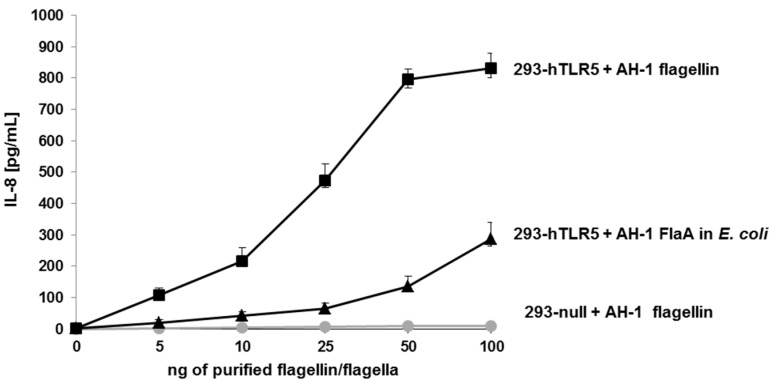
IL-8 production of HEK293-null and HEK293-hTLR5 cells. HEK293-null and HEK293-hTLR5 cells were stimulated with five different increased amounts (from 5 to 100 ng) of purified polar flagella from *A. hydrophila* AH-1 (wild-type) strain. HEK293-hTLR5 cells were also stimulated with *A. hydrophila* AH-1 polar flagellin FlaB obtained in *E. coli* (non-glycosylated). Data shown are means ± SD of three independent experiments.

When we stimulated HEK293-null cells (control) with purified *A. hydrophila* AH-1 polar flagellins, no IL-8 production was observed (Figure 7).

The degree of TLR5 activation was studied through the production of IL-8 using the HEK293 cell line, which was stably transfected with TLR5. HEK293-hTLR5 cells stimulated with purified *A. hydrophila* AH-1polar flagellins (wild type) showed varying levels of IL-8 production in agreement with the amount of flagellin used (Figure 7). However, HEK293-hTLR5 cells were stimulated with identical amounts of *A. hydrophila* AH-1 polar flagellin FlaB unglycosylated monomer obtained in *E. coli*, and the amount of IL-8 production was reduced (60%).

## 3. Discussion

In the current study, we demonstrated that *A. hydrophila* strain AH-1 (serotype O11) flagella, the constitutively expressed polar flagellum but not the inducible peritrichous lateral flagella, showed modification with a putative *O*-linked glycan moiety. As previously published, strain AH-3 (serotype O34) showed both flagella glycosylated. Both serotypes are among the four dominant serotypes (O11, O16, O18, and O34) that are associated with gastroenteritis and septicemia in clinical studies [26]. Mass spectrometry fragmentation of the putative glycan provides some evidence that the sugar contains similarities to previously characterized pseudaminic acid-like sugars. The mass spectrometry data and putative elemental formula suggest the presence of two methyl groups and two molecules of water, and an unknown mass of 25 Da.

The altered or incomplete flagellin glycosylation resulted in altered motility phenotypes. This was also observed with *Clostridium difficile*, where the abolition of flagellin glycosylation resulted in the loss of motility [27]. Studies of *A. hydrophila* strain AH-3 showed that deletion mutants of pseudaminic acid biosynthesis abolish polar and lateral flagellum formation [23]. However, deletion mutants of pseudaminic acid biosynthesis in strain AH-1 (serotype O11) abolish polar flagellum formation but not lateral flagella. Because the presence of the putative pseudaminic acid seems to be a requirement for flagellin export and flagella formation in *Aeromonas* [23], this point is in agreement with our results that lateral flagella in this strain AH-1 are not glycosylated.

AH-1Δ*rmlB* mutant is unable to produce O11-antigen LPS [18]; however, it is able to produce either polar or lateral flagella under induced conditions by TEM studies. No interaction between the flagella *O*-glycosylation and *O*-antigen LPS biosynthetic pathway was found in *A. hydrophila*. This is in contrast with studies of *Pseudomonas aeruginosa*, which suggested involvement of the *O*-antigen biosynthesis in *O*-glycosylation [28]. In addition, *O*-antigen LPS and flagella have been described as important molecules for bacterial adherence or biofilm formation in *Aeromonas* [17].

Results from the current study suggest that flagella is a more important adhesion factor than *O*-antigen LPS, as shown by adhesion to Hep-2 cells. Furthermore, the polar flagellum seems to be a determinant factor, with the lateral flagella unable to fully compensate for lack of expression of polar flagellin. This is the first study to show that polar flagellum glycosylation in *Aeromonas* is a determinant factor in adherence to eukaryotic cells. The results obtained in biofilm formation studies are more marked, with polar flagellum a requirement for biofilm formation, with mutants deficient in polar flagellin expression unable to form biofilms. Then, if there is any compensation from the lateral flagella to the loss of polar flagellum, it is unable to achieve the degree for biofilm formation. The *O*-antigen LPS mutant, able to produce either polar or lateral flagellin, was observed to form biofilms, but in a reduced capacity.

Toll-like receptors (TLRs) are major components of innate immunity. TLR5 is involved in recognizing bacterial flagellin and, after binding, it triggers the induction of pro-inflammatory cytokines such as IL-8 by the myeloid differentiation primary response gene 88 (MyD88)-dependent signaling pathway [29]. The results obtained indicate that *A. hydrophila* AH-1 polar flagellin glycosylation is important for and quantitatively related to immune stimulation of IL-8 production via TLR5. A clear reduction in IL-8 production was observed when polar non-glycosylated flagellin monomers (FlaB) were expressed in *E. coli*. Fish infected with pathogenic bacteria showed a significant enhanced IL-8 expression in the blood and intestine [30]. It is tempting to speculate the possible role of *A. hydrophila* AH-1 polar flagella glycosylation in cell invasion as observed for different *A. hydrophila* wild-type strains [17].

This study supports the existence of a complex mechanism of flagella glycosylation in different *A. hydrophila* strains. We show for the first time the presence of inducible lateral flagella, either with lateral flagellins glycosylated or not, in different *A. hydrophila* strains. We also show that different *A. hydrophila* strains used different glycans for polar flagellin glycosylation. Furthermore, we continue to shed light on the biological role of flagellum glycosylation, showing their implication in flagellum production, motility, adhesion to eukaryotic cells, biofilm formation, and TLR5 activation.

## 4. Experimental Section

### 4.1. Bacterial Strains, Plasmids, and Growth Conditions

*E. coli* strains were grown on Luria-Bertani (LB) Miller broth and LB Miller agar at 37 °C, while *Aeromonas* strains were grown either in tryptic soy broth (TSB) or agar (TSA) at 30 °C. When indicated, kanamycin (50 μg/mL), rifampicin (100 μg/mL), spectinomycin (50 μg/mL), and chloramphenicol (25 μg/mL) were added to the media.

The bacterial strains and plasmids used in this study are listed in Table 3.

**Table 3:** Bacterial strains and plasmids used.

Strain or Plasmid	Relevant Characteristics	Reference or Source
*E. coli* Strains		
DH5α	F^−^ *end A hsdR17* (rK^−^ mK^+^) *supE44 thi-1 recA1 gyr-A96 Ф80lacZ*M15	[31]
XL1-Blue	*recA1 endA1 gyrA96 thi-1 hsdR17 supE44 relA1 lac* (F^−^ p*roABlacI*q*Z*_M15 Tn*10*)	Stratagene
BL21(λD3)	F^−^ *ompT* *hsdS_B_* (r_B_^−^ m_B_^−^) *gal* *dcm*(λD3)	Novagen
***A. hydrophila* Strains**		
AH-1	O11, Wild type	[19]
AH-Rif ^R^	AH-1, spontaneous rifampicin resistant mutant, Rif ^R^	[19]
AH-1Δ*flaB*-*J*	AH-1 mutant in frame unable produce polar flagellum but able to produce lateral flagella	This study
AH-1Δ*rmlB*	AH-1 mutant in frame unable produce O11-antigen LPS	[18]
AH-1Δ*pseB*	AH-1 *pseB* mutant in frame with pDM4	This study
AH-1Δ*pseI*	AH-1 *pseI* mutant in frame with pDM4	This study
**Plasmids**		
pRK2073	Helper plasmid,,Spc ^R^	[32]
pBAD33	arabinose inducible expression vector, Cm ^R^	[33]
pBAD-*pseB*	pBAD33 with AH-1 *pseB*	This study
pBAD-*pseI*	pBAD33 with AH-1 *pseI*	This study
pDM4	*pir* dependent with *sacAB* genes, oriR6K, Cm ^R^	[34]
pDM4-*flaB*-*J*	pDM4 with AH-1 *flaB-J* fragment, Cm ^R^	This study
pDM4-*pseB*	pDM4 with AH-1 *pseB* fragment, Cm ^R^	This study
pDM4-*pseI*	pDM4 with AH-1 *pseI* fragment, Cm ^R^	This study
pET-30 Xa/LIC	IPTG inducible expression vector Km ^R^	Novagen
pET-30-FlaB-AH1	pET-30 Xa/LIC with *A. hydrophila* AH-1 *flaB*	This study

^R^, resistance.

### 4.2. DNA Techniques

Standard procedures [35,36,37,38,39] were used as previously described [21,22].

### 4.3. Construction of Defined Mutants

The chromosomal in-frame *flaB-J*, *pseB*, *pse*I and *rmlB* deletion mutants *A. hydrophila* AH-1∆*flaB*-*J*, AH-1∆*pseB*, and AH-1Δ*pseI*, respectively, were constructed by allelic exchange as described by Milton *et al.* [34]. Briefly, upstream (fragment AB) and downstream (fragment CD) of *flaB-J*, *pseB*, and *pseI* were independently amplified using two sets of asymmetric PCRs. Primer pairs A-FlaB (5′-CGGGATCCAACAGTCTGCCAATGGTTC-3′) and B-FlaB (5′-CCCATCCACTAAACTTAAACAGTTAG CCTGAGCCAAAATG-3′) and C-FlaJ (5′-TGTTTAAGTTTAGTGGATGGGAGACA ACAGCTAGGGGAGTT-3′) and D-FlaJ (5′-CGGGATCCAACGTTTCACAAGCAAGA-3′) amplify DNA fragments of 581 (AB) and 637 (CD) bp for *flaB-J* in-frame deletion. Primer pairs A-PseB (5′-GAAGATCTGAGGACAAACAACGGATG-3′) and B-PseB (5′-CCCATCCACTAAACTTAAACATGTCTTGACCAGCATCTT-3′) and C-PseB (5′-TGTTTAAGTTTAGTGGATGGGATGAACCAGCAGGTGTTGT-3′) and D-PseB (5′-GAAGATCTAAGCTGAAGACCGTCATGT-3′) amplify DNA fragments of 759 (AB) and 574 (CD) bp for *pseB* in-frame deletion. Primer pairs A-PseI (5′-CGGGATCCAATGCTGGATGATGAGCAA-3′) and B-PseI (5′-CCCATCCACTAAACTTAAACAGTCAGCGGTATAGGTTTGCA-3′) and C-PseI (5′-TGTTTAAGTTTAGTGGATGGGAGACGAGGCAAAGCAGTC-3′) and D-PseI (5′-CGGGATCCTTTAACTGGCCTGGCTCTA-3′) amplify DNA fragments of 764 (AB) and 630 (CD) bp for *pseI* in-frame deletion. We use plasmid pDM4 [22] to obtain *A. hydrophila* AH-1Δ*flaB*-*J*, AH-1Δ*pseB*, and AH-1Δ*pseI* mutants as previously described for other mutants [40].

### 4.4. Plasmid Constructions

Plasmids pBAD-*pseB* and pBAD-*pseI* containing the complete *pseB* and *pseI* of *A. hydrophila* AH-1 under the arabinose promoter (pBAD) on pBAD33 [33] were obtained. Oligonucleotides 5′-GCTCTAGATGGAATAAAACTGGCATCA-3′ and 5′-CCAAGCTTGACCTTGGGTCAGATAATCA-3′ generated a band of 1198 bp containing the *pseB*; Oligonucleotides 5′-TCCCCCGGGTTCACTTTTCACGCCTAT-3′ and 5′-GCTCTAGACTAATGCTAAAGCGACAACG-3′ generated a band of 1880 bp containing the *pseI*. The construction of the pBAD-*pseB* and pBAD-*pseI* plasmids was done as previously described for other genes [40]. The plasmids were introduced independently into the *E. coli* DH5α [31].

### 4.5. Flagella Purification

*A. hydrophila* AH-1 was grown in TSB for the polar flagellum purification and in TSA for the isolation of lateral flagella, and the flagella purified as previously described [40]. Purified flagella were analyzed by SDS-PAGE or by glycosylation chemical studies.

### 4.6. Motility Assays (Swarming and Swimming)

The assays were performed as previously described [21,22].

### 4.7. Transmission Electron Microscopy (TEM)

TEM was performed on Formvar-coated grids and negative stained with a 2% solution of uranyl acetate pH 4.1.

### 4.8. Electrospray Liquid Chromatography Mass Spectrometry Analysis of Intact Flagellins

Mass spectrometry studies of intact flagellin proteins were carried out using 50 μL aliquots of protein-containing sample as described previously, with some modification [23]. The purified flagellin preparations were injected onto a protein microtrap (Michrom Bioresources Inc., Auburn, CA, USA) connected to a gradient HPLC pump (Agilent 1100 HPLC, Agilent Technologies, Santa Clara, CA, USA). Solvent A was 0.1% formic acid in HPLC-grade water (Fisher, Waltham, MA, USA) and solvent B was 0.1% formic acid in acetonitrile. An HPLC gradient of 5%–60% solvent B (1 mL/min) over 60 min was used to resolve the protein mixture. A pre-column splitter was used to direct 35 μL/min of the HPLC column eluate into the electrospray interface of the QTOF2 (Waters, Milford, MA, USA), allowing real-time monitoring of ion elution profiles. Intact masses of proteins were calculated by spectral deconvolution, using MaxEnt (Waters, Beverly, MA, USA). For each protein, front-end collision-induced dissociation (feCID) was performed by increasing the cone voltage from 45 to 85 V and glycan-associated oxonium ions were observed. Tandem MS of prominent ions were also performed. In addition, multiply charged protein ions were selected for MS/MS. The collision energy was increased from X to Y incrementally and labile-, protein-, and glycan-associated fragment ions were observed.

### 4.9. Solution Enzymatic Digests and Bottom-Up Mass Spectrometry Analysis of Glycopeptides

In preliminary experiments, tryptic digests of intact proteins were performed, with 50 to 200 μg of pure flagellin prepartion in solution digested with trypsin (Promega, Madison, WI, USA) at a ratio of 30:1 (protein/enzyme, *w*/*w*) in 50 mM ammonium bicarbonate at 37 °C overnight. Protein digests were analyzed by nano-liquid chromatography MS/MS (nLC-MS/MS) using either a Q-TOF Ultima hybrid quadrupole time-of-flight MS (Waters) or an LTQ XL orbitrap MS (Thermo Fisher Scientific, Ottawa, ON, Canada) coupled to a nanoAcquity ultrahigh-pressure liquid chromatography system (Waters). MS/MS spectra were acquired automatically on doubly, triply, and quadruply charged ions in collision-induced dissociation (CID) mode for initial glycopeptide identification. Peak lists were automatically generated by proteinlynx software (Waters) with the following parameters: smoothing—four channels, two smooths, Savitzky-Golay mode; centroid—minimum peak width at half height of four channels, centroid top 80%. Tryptic peptides were analyzed by nano-LC-MS/MS, and spectra were searched against the National Centre for Biotechnology nonredundant database and an in-house database of sequenced flagellin proteins using mascot 2.0.6 (Matrix Science, London, UK). A peptide score of 30 and above for a top-ranked hit was taken as positive identification, with each MS/MS spectrum verified by manual inspection. MS/MS spectra not identified by MASCOT were *de novo* sequenced. Proteinase K digests were analyzed up nanoliquid chromatography MS/MS, using a Q-TOF Ultima, coupled to a nanoAcquity ultrahigh-pressure liquid chromatography system (Waters, Milford, MA, USA). The column setup and gradients did not deviate from those reported in our other studies [23]. MS/MS spectra were acquired automatically on doubly, triply, and quadruply charged ions in collision-induced dissociation (CID) mode for initial glycopeptide identification. High resolution multi-stage mass spectrometry studies of glycan moieties were performed in high resolution (100,000) mode on the LTQ XL Orbitrap Mass spectrometer (Fisher Scientific, Waltham, MA, USA). All proteinaseK MS/MS spectra were *de novo* sequenced, no database searching was employed in peptide sequence assignment.

### 4.10. Adherence Assay to HEp-2 Cell

The adherence assay was conducted in triplicate as previously described [40].

### 4.11. Biofilm Formation

Quantitative biofilm formation was performed in a microtiter plate as described previously [40], by adapting the method of Pratt and Kotler [41].

### 4.12. Purification of A. hydrophila AH-1 His_6_-FlaB

For *flaB* overexpression the pET-30 Xa/LIC vector (Novagen, Nottingham, UK) and AccuPrime (Invitrogene, Madrid, Spain). High-fidelity polymerase was used. The *A. hydrophila* AH-1 *flaB* was amplified from genomic DNA using primers PET-A3flaB-for 5′-GGTATTGAGGGTCGCATGCTTGCTGTGTGTTTACC-3′ and PET-A3flaB-rev 5′-AGAGGAGAGTTAGAGCCATTCCTTTCTTCCCAAAGC-3′, and the PCR product ligated into pET-30 Xa/LIC (Novagen), and electroporated into *E. coli* BL21(*λ*DE3). The His_6_-FlaB protein was overexpressed and cell lysates obtained as previously reported for other proteins [42].

### 4.13. Interleukin-8 (IL-8) Assay with Human Embryonic Kidney Cells

IL-8 concentration using HEK293-hTLR5 and HEK293-null cells was performed as previously described [40].

### 4.14. Statistical Analysis

Results are expressed together with the standard deviations (SD) from several experiments and Student’s *t*-test was used to compare mean values. Differences were considered significant when *p*-values were <0.05.
